# Cardiomyopathy is common in patients with the mitochondrial DNA m.3243A>G mutation and correlates with mutation load

**DOI:** 10.1016/j.nmd.2012.03.001

**Published:** 2012-07

**Authors:** Kieren G. Hollingsworth, Grainne S. Gorman, Michael I. Trenell, Robert McFarland, Robert W. Taylor, Douglass M. Turnbull, Guy A. MacGowan, Andrew M. Blamire, Patrick F. Chinnery

**Affiliations:** a Newcastle Magnetic Resonance Centre, Institute of Cellular Medicine, Newcastle University, Campus for Ageing and Vitality, NE4 5PL, UK; b Mitochondrial Research Group and NIHR Biomedical Research Centre for Ageing and Age-related Disease, Institute for Ageing and Health, Newcastle University, Newcastle upon Tyne, NE2 4HH, UK; c Department of Cardiology, Freeman Hospital, Newcastle upon Tyne, NE7 7DN and Institute of Genetic Medicine, Newcastle University, UK; d Institute of Genetic Medicine, Newcastle University, Newcastle upon Tyne, NE2 4HH, UK

**Keywords:** Mitochondrial disease, Cardiomyopathy, Tagging, MRI

## Abstract

Although neuromuscular clinical features often dominate the clinical presentation of mitochondrial disease due to the m.3243A>G mitochondrial DNA (mtDNA) mutation, many patients develop cardiac failure, which is often overlooked until it reaches an advanced stage. We set out to determine whether cardiac complications are sufficiently common to warrant prospective screening in all mutation carriers. Routine clinical echocardiography and 3 Tesla cardiac MRI were performed on ten m.3243A>G mutation carriers and compared to age and gender matched controls, with contemporaneous quadriceps muscle biopsies to measure respiratory chain activity and mtDNA mutation levels. Despite normal echocardiography, all ten m.3243A>G mutation carriers had evidence of abnormal cardiac function on MRI. The degree of cardiac dysfunction correlated with the percentage level of mutant mtDNA in skeletal muscle. Sub-clinical cardiac dysfunction was a universal finding in this study, adding weight to the importance of screening for cardiac complications in patients with m.3243A>G. The early detection of cardiac dysfunction with MRI opens up opportunities to prevent heart failure in these patients through early intervention.

## 1 Introduction

Neurological features usually dominate the clinical presentation of patients harbouring the m.3243A>G mitochondrial DNA (mtDNA) mutation; with migraine, encephalopathy, seizures, stroke-like episodes, deafness, ophthalmoplegia and myopathy all being common. Although well recognised in advanced cases [1], cardiomyopathy is often overlooked, despite being a major cause of morbidity and mortality [2,3], and it may explain the increased rate of sudden unexpected death observed in Finnish patients harbouring m.3243A>G [4]. Using relatively insensitive 2D-echocardiography, left-ventricular hypertrophy was been detected in 56% of cases [2], raising the possibility that cardiac dysfunction is major feature of the disorder potentially amenable to early treatment to prevent cardiac failure [5]. To address this issue, we used cardiac magnetic resonance imaging (MRI) to determine the prevalence of cardiac dysfunction in m.3243A>G mutation carriers, with contemporaneous genetic and biochemical studies on skeletal muscle to elucidate the mechanism.

## 2 Methods

We studied ten m.3243A>G mutation carriers (five male, five female, mean age 42.5 ± SD 9 years). Tables 1 and 2 shows the clinical and laboratory data, including co-morbidities and medication), and sixteen healthy age matched controls (eight male, eight female, mean age 41.6 ± SD 15 years). No significant abnormalities were found on electrocardiography (ECG) or 2D-echocardiography in either group. Systolic and diastolic blood pressure was normal. Nine m.3243A>G mutation carriers underwent a left vastus lateralis skeletal muscle needle biopsy. Respiratory chain complex activities, and the level of m.3243A>G mutation were determined in each biopsy as described [6], along with the mutation load in urinary epithelium and blood when available.

3-Tesla ECG-gated cardiac MRI was performed using a six-channel cardiac coil on supine subjects (Philips Intera Achieva, Best, NL). Structural evaluation included left ventricular (LV) mass relative to body surface area, ejection fraction, end-systolic and end-diastolic volumes. The eccentricity ratio was calculated as the ratio of LV mass to end-diastolic volume and an increase in this parameter was interpreted as evidence of concentric remodelling. Cardiac tagging was used to evaluate function measured as torsion and regional myocardial strains: these include the torsion-to-endocardial strain ratio, a measure of the relative contribution of the endocardial and epicardial contractions to systole; and radial thickening, the percentage increase in average wall thickness from end-diastole to systole (Detailed MRI methods described on-line). Written informed consent was obtained from all participants and institutional ethics was obtained.

## 3 Results

**Fig. 1:**
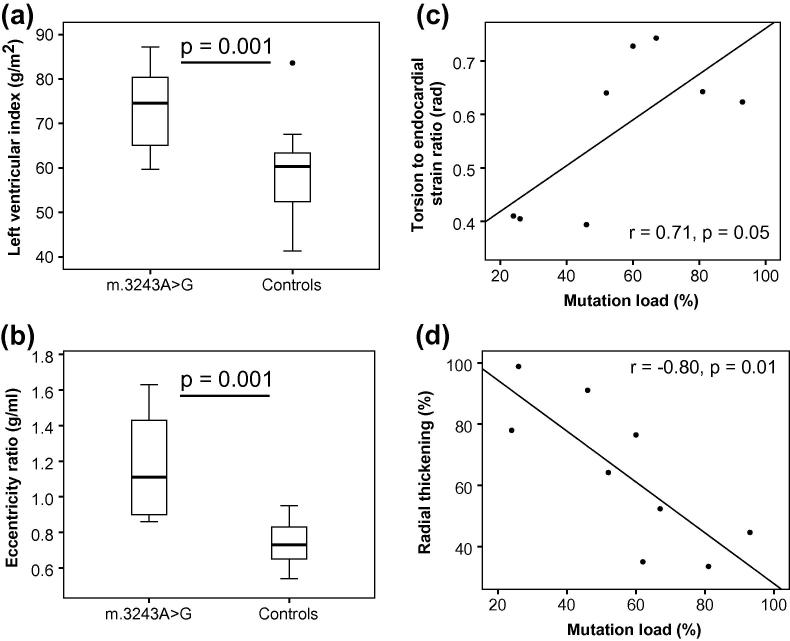
For m.3243A>G and control groups – (a) left ventricular index (left ventricular mass/body surface area), (b) eccentricity ratio. Within the m.3243A>G group, mutation load correlates with (c) torsion to endocardial strain ratio and (d) radial thickening.

All m.3243A>G patients had structural and functional cardiac abnormalities on MRI when compared to age-matched controls (Table 3. Online videos). These included, increased LV index (74 g/m^2^ vs. 59 g/m^2^, *p* = 0.001), reduced end systolic (36 ml vs. 61 ml, *p* = 0.0005) and diastolic volume (103 ml vs. 143 ml, *p* = 0.002), increased peak torsion (7.3° vs. 6.0° , *p* = 0.02) and an increased eccentricity ratio (1.19 g/ml vs 0.74 g/ml, *p* = 0.001, Fig. 1), indicative of cardiac remodelling. The percentage level of m.3243A>G mutation in skeletal muscle correlated with the decrease in respiratory chain complex I activity (*κ* = 0.71, *p* = 0.01); and both muscle mutation load and complex I activity correlated with the torsion to endocardial strain ratio (*κ* = 0.71, *p* < 0.05 and *κ* = −0.73, *p* < 0.03, respectively), and with reduced radial thickening (*κ* = −0.80, *p* = 0.01, Fig. 1).

## 4 Discussion

We found structural and functional cardiac abnormalities in all of the m.3243A>G patients we studied. The major findings were: (1) concentric left ventricular hypertrophy in the absence of systemic hypertension; and (2) a re-orientation of myocardial strains with reduced longitudinal shortening and increased torsion. These findings indicate that sub-clinical cardiomyopathy is common in m.3243A>G patients, and may be a universal finding. Although the majority of subjects in our study had clinical features of mtDNA disease, one was an asymptomatic carrier who also had abnormal cardiac function on MRI. Given previous reports of progressive cardiac hypertrophy leading to irreversible dilated cardiomyopathy in m.3243A>G patients [7], our observations re-enforce the importance of cardiac monitoring in all m.3243A>G mutation carriers. Cardiac MRI provides a sensitive method of detecting early cardiac dysfunction, complementing echocardiography. Early intervention may reduce cardiac remodelling in m.3243A>G patients and thus delay left ventricular failure, although this needs to be prospectively studied.

Despite known differences in tissue mutation load, we observed a correlation between the percentage level of m.3243A>G and complex I activity in skeletal muscle, and several key cardiac measures of early cardiac dysfunction. When taken together, these observations support a central role for the genetic and biochemical defect in the pathogenesis of the cardiomyopathy, rather than an indirect mechanism through reduced physical fitness or co-morbidity related to the diabetes known to occur in m.3243A>G carriers.

It remains intriguing that the two lowest muscle mutation levels were observed in a 31 year old patient without symptoms (m.3243A>G = 24%) and in a 46 year old patient with a severe MELAS phenotype (m.3243A>G = 26%). These findings reflect the known poor correlation between muscle mutation load and neurological phenotype; and contrast with the correlation that we observed between skeletal muscle mutation load and the degree of cardiac dysfunction. By inference, this implies that mutation load in myocardium is closely related to mutation load in skeletal muscle, and that the consequences of a given mutation level are similar in both cardiac and skeletal muscle. The same does not appear to be the case for the central nervous system, where there is a much more diverse range of cell types of different embryological origins, each responding to a given level of m.3243A>G to differing degrees, and contributing to the combined neurological phenotype in different ways. The lack of any correlation between blood mutation levels and the cardiac phenotype is not surprising, given that m.3243A>G in blood decreases with age [8], unlike other tissues [9].

Our observations also suggest that measures aimed at reducing mutation load or improving complex I activity are likely to be beneficial for the cardiomyopathy in m.3243A>G patients. Although there are currently no known methods of reducing cardiac mutation load, it is conceivable that “gene shifting” techniques currently being explored in skeletal muscle could be of benefit [10]. This would involve the delivery or local activation of cardiomyocyte precursors potentially harbouring a lower mutation load. An alternative approach could involve drugs known to increase mitochondrial biogenesis which could improve both the genetic and biochemical defect in a similar way to aerobic exercise in skeletal muscle [11]. Interestingly, we saw no correlation between m.3243A>G mutation load, complex I activity, or cardiac dysfunction and the specific enzyme activities of complex II and citrate synthase (Table 2). These enzymes are considered to be markers of mitochondrial proliferation and biogenesis, suggesting that compensatory mitochondrial proliferation had not been activated in the skeletal muscle of the patients described here, despite the complex I defect. Finally, treatment with anti-oxidant drugs provides hope of treatment in the short term. The anti-oxidant properties of idebenone may explain the beneficial effects of this drug on the cardiomyopathy in Friedreich’s ataxia, which also has a mitochondrial basis. The approach we describe here provides an objective method for assessing the efficacy of novel treatments in patients with m.3243A>G. This will hopefully lead to the first cardioprotective therapy for primary mitochondrial disease.

## Figures and Tables

**Table 1:** Clinical data for the patients harbouring m.3243A>G. It was not possible to obtain a muscle biopsy from subject 10.

No.	Age (years)	Sex	Clinical features	Medication	Echo LVEF (%)
1	55	M	Deafness, diabetes, ht, ha	ACEi, a, m, In, St	66
2	48	M	Deafness, diabetes, ha	ACEi, St, In	70
3	46	M	Deafness, hc, seizures, SLEs.	c, a, ACEi, St	55
4	34	F	Deafness, constipation, ha	–	>60
5	44	M	Migraine, constipation	f, a	61
6	42	F	Deafness, diabetes, ht, SLEs	z, ACEi	>55
7	55	F	Constipation, ha; asthma	–	>55
8	30	F	Deafness, diabetes, ht, hcl, SLEs.	pr, St, c, In, le	>60
9	31	M	None	–	60
10	40	F	Deafness, diabetes	–	n/a

*Key:* ha = headaches, hc = hypercholesterolaemia, ht = hypertension, ACEi = angiotensin converting enzyme inhibitor, St = statin ± ezetmibe, ln = insulin, a = aspirin, m = metformin, c = carbamazepine, f = flunarazine, z = Na val, pr = propanolol, le = levemenre. SLEs: stroke-like episodes.

**Table 2:** Molecular genetic and biochemical data for the patients harbouring m.3243A>G. The percentage level of m.3243A>G and respiratory chain complex activities were measured as previously described.

No.	3243A>G mutation load			I/mg	II/mg	III/mg	IV/mg	CS/mg
	SKM	UE	BLD					
1	62	66	nd	0.623	1.017	5.613	7.891	6.393
2	81	nd	nd	0.284	0.881	8.185	4.966	7.128
3	26	82	28	1.354	2.061	17.294	21.440	9.116
4	67	80	26	0.645	3.237	21.716	17.507	11.628
5	46	48	5	0.626	1.344	8.861	9.589	6.421
6	52	nd	nd	1.176	4.710	34.082	28.429	21.743
7	60	20	9	1.126	2.367	14.938	19.049	13.412
8	93	86	3	0.207	1.807	13.000	4.939	18.911
9	24	34	7	1.530	2.155	16.711	16.024	12.584
10	73	57	21	–	–	–	–	–

The percentage level of m.3243A>G and respiratory chain complex activities were measured as previously described. It was not possible to obtain a muscle biopsy from subject 10. CS = citrate synthase activity, nd = not determined. SKM = skeletal muscle, UE = urinary epithelium, BLD = blood.

**Table 3:** Cardiac morphology and function parameters for controls and mutation carriers. LV = left ventricle.

Parameter	Controls	m.3243A>G	*p* value
Age (years)	41.6 ± 15	42.5 ± 9	ns
Heart rate (bpm)	61 ± 13	71 ± 12	ns
Systolic blood pressure (mm Hg)	128 ± 13	130 ± 14	ns
Diastolic blood pressure (mm Hg)	76 ± 10	83 ± 10	ns
Height (cm)	171 ± 11	165 ± 11	ns
Weight (kg)	75 ± 10	63 ± 16	0.02
Body Surface Area (BSA, m^2^)	1.8 ± 0.1	1.6 ± 0.2	ns

*LV mass and global systolic function*			
LV mass (g)	106.7 ± 20.4	119.7 ± 29.6	ns
LV index [LV mass/BSA] (g/m^2^)	59.3 ± 9.5	73.7 ± 9.9	0.001
Stroke volume (ml)	82 ± 15	67 ± 16	0.02
End-diastolic volume (ml)	143.0 ± 32.0	103.1 ± 23.3	0.002
End-systolic volume (ml)	61.0 ± 18.7	36.2 ± 10.8	0.0005
Ejection fraction (%)	58 ± 5	65 ± 6	0.004
Eccentricity ratio [LV mass/EDV] (g/ml)	0.74 ± 0.11	1.19 ± 0.30	0.001

*LV strains*			
Longitudinal shortening (%)	18.1 ± 2.8	14.5 ± 4.0	0.01
Peak torsion (degrees)	6.0 ± 1.4	7.3 ± 1.3	0.02
Peak circumferential strain (%)	17.8 ± 2.7	17.5 ± 2.7	ns
Radial thickening (%)	59 ± 20	65 ± 23	ns
Torsion to endocardial strain ratio (rad)	0.49 ± 0.13	0.57 ± 0.14	ns

## Appendix A Supplementary data

Supplementary data 1 Structural Cardiac Magnetic Resonance Cine Imaging.
