# Supplementary material for: Cardiomyopathy is common in patients with the mitochondrial DNA m.3243A>G mutation and correlates with mutation load
Source: Neuromuscul Disord. 2012 Jul;22-334(7):592–6. doi: 10.1016/j.nmd.2012.03.001 (PMC3387369; doi:10.1016/j.nmd.2012.03.001)
Supplement: Supplementary data 1 — Structural Cardiac Magnetic Resonance Cine Imaging. [file mmc1.doc]

***Supplementary methods on-line***

***Structural Cardiac Magnetic Resonance Cine Imaging***

Cardiac examinations were performed using a 3T Philips Intera Achieva scanner (Best, NL). A dedicated 6-channel cardiac coil (Philips, Best, NL) was used with the subjects in a supine position and electrocardiogram (ECG) gating (Philips vectorcardiogram, VCG system). Cardiac magnetic resonance cine imaging was acquired to assess cardiac morphology, and systolic function. A stack of balanced steady-state free precession images was obtained in the short axis view during breath holding covering the entire left ventricle (TR/TE/FA/NEX = 3.7ms/1.9ms/40^o^/1, FOV = 350mm turbo factor 17, slice thickness 8mm, 0mm gap, 14 slices, 25 phases, resolution 1.37x1.37mm, temporal duration approx. 40ms per phase, dependent on heart rate). Image analysis was performed using the cardiac analysis package of the ViewForum workstation (Philips, Best, NL). Manual tracing of the epicardial and endocardial borders was performed on the short axis slices at end-systole and end-diastole. The contours were reviewed by viewing the cine data with the contours attached. The basal slice selected for analysis at end-diastole and at end-systole occurred when at least fifty percent of the blood volume was surrounded by the myocardium.[^2^](#_ENREF_2) The apical slice was defined as the last slice showing intra-cavity blood pool. Papillary muscles were included in calculations of mass but excluded from calculations of volume. The interventricular septum was included as part of the left ventricle. Left ventricular mass (LV mass), ejection fraction, end-systolic and end-diastolic volumes were calculated. Myocardial mass was determined by multiplying the tissue volume by1.05 g/cm^3^ (specific density of myocardium). The body surface area was estimated from the subjects’ weight and height according to the formula of Dubois and Dubois [^3^](#_ENREF_3) and this was used to standardize the measurements for subject size (denoted by the suffix “index”). The eccentricity ratio was calculated as the ratio of LV mass to end-diastolic volume and an increase in this parameter was interpreted as evidence of concentric remodeling.

***Cardiac tagging***

Tagged images of the myocardium in the short axis were obtained at the same session as the morphological imaging using the same cardiac coil. A multi-shot turbo-field echo sequence was used (TR/TE/FA/NEX = 4.9/3.1/10^o^/1, turbo factor 9, SENSE factor 2, FOV 350x350mm, voxel size 1.37x 1.37mm with an orthogonal CSPAMM grid[^4^](#_ENREF_4) with tag spacing of 7mm, 12 phases). Two adjacent short-axis slices of 10mm thickness were acquired at mid-ventricle with a 2mm gap. The Cardiac Image Modeling package (University of Auckland) was used to analyze the tagging data by aligning a mesh on the tags between the endo- and epi-cardial contours. Circumferential strain and the rotation of the two planes were calculated throughout the cardiac cycle. The torsion between the two planes (taken as the circumferential-longitudinal shear angle) was calculated as previously described [^5^](#_ENREF_5) to account for the radius of the ventricle. In the healthy heart, torsion occurs such that there is homogeneity of fibre shortening across the myocardial wall and is a marker of the dominance of epicardial fibres over endocardial fibres as a consequence of the greater radius in the epicardium. This can be quantified by a ratio of the peak torsion (in radians), defined as the shear angle between two planes on the epicardial surface,[^6^](#_ENREF_6) and the peak circumferential strain in the endocardial third of the myocardium and is referred to as the torsion to endocardial strain ratio. This ratio has been shown to be near constant amongst healthy subjects, and to increase with both healthy ageing and disease. It was not possible to analyze the circumferential strain or torsion data for subject 1 due to patient motion during tagging. The repeatability of the cardiac tagging analysis had been previously assessed using a Bland Altman analysis six healthy subjects with no history of cardiac disease and the repeatability of the torsion to endocardial strain ratio found to be 0.07 radian, for torsion 0.8^o^ and 1.2% for peak circumferential strain measurements. Longitudinal shortening was determined from cine-MRI in the 4-chamber view by determining the perpendicular distance from the plane of the mitral valve to the apex in systole and diastole, and expressing the difference in the measures as a percentage of the diastolic value. The myocardial wall thicknesses at diastole and diastole were determined from the standard imaging at the same level as the cardiac tagging by averaging the distance between the epicardial and endocardial contours around the left ventricle. The percentage increase (radial thickening) from diastole to systole was also calculated.

***Legend to supplementary videos***

**Video 1:** Mid-ventricular short axis cine video of an m.3243A>G mutation carrier, demonstrating left ventricular hypertrophy and reduced end-systolic and end-diastolic volumes (video at 50% real-life speed for clarity).

**Video 2:** Mid-ventricular short axis cine video of a healthy control subject with heart mass and normal end-systolic and end-diastolic volumes (video at 50% real-life speed for clarity).

**Supplementary references**

1. Kirby DM, Thorburn DR, Turnbull DM, Taylor RW. Biochemical assays of respiratory chain complex activity. Methods Cell Biol. 2007; 80: 93-119.

2. Hudsmith LE, Petersen SE, Francis JM, Robson MD, Neubauer S. Normal human left and right ventricular and left atrial dimensions using steady state free precession magnetic resonance imaging. Journal of Cardiovascular Magnetic Resonance 2005;7:775-782.

3. Dubois D, Dubois EF. Nutrition metabolism classic - a formula to estimate the approximate surface-area if height and weight be known (reprinted from archives internal medicine, vol 17, pg 863, 1916). Nutrition 1989;5:303-311.

4. Fischer SE, McKinnon GC, Maier SE, Boesiger P. Improved myocardial tagging contrast. Magnetic Resonance in Medicine 1993;30:191-200.

5. Buchalter MB, Weiss JL, Rogers WJ, et al. Noninvasive quantification of left-ventricular rotational deformation in normal humans using magnetic-resonance-imaging myocardial tagging. Circulation 1990;81:1236-1244.

6. Lumens J, Delhaas T, Arts T, Cowan BR, Young AA. Impaired subendocardial contractile myofiber function in asymptomatic aged humans, as detected using MRI. American Journal of Physiology-Heart and Circulatory Physiology 2006;291:H1573-H1579.

7. Van der Toorn A, Barenbrug P, Snoep G, et al. Transmural gradients of cardiac myofiber shortening in aortic valve stenosis patients using MRI tagging. American Journal of Physiology-Heart and Circulatory Physiology 2002;283:H1609-H1615.
