# Supplementary figures and images for: Cardiomyopathy is common in patients with the mitochondrial DNA m.3243A>G mutation and correlates with mutation load
Source: Neuromuscul Disord. 2012 Jul;22-334(7):592–6. doi: 10.1016/j.nmd.2012.03.001 (PMC3387369; doi:10.1016/j.nmd.2012.03.001)

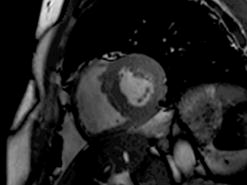

Supplement: Supplementary video 2 [file mmc2.jpg]
